# Effect of Periapical Diseases in Development of MRONJ in Immunocompromised Mouse Model

**DOI:** 10.1155/2019/1271492

**Published:** 2019-09-22

**Authors:** Nian Jing Rao, Ru Qing Yu, Jing Yi Wang, Alexandra Helm, Li Wu Zheng

**Affiliations:** ^1^Discipline of Oral and Maxillofacial Surgery, Faculty of Dentistry, The University of Hong Kong, Hong Kong SAR, China; ^2^Department of Medicine and Orofacial Surgery, Faculty of Dentistry, Complutense University of Madrid, Madrid, Spain

## Abstract

**Objectives:**

This study aimed to assess the effect of zoledronic acid on an immunocompromised mice model with periapical disease.

**Materials and Methods:**

Thirty C57BL/6N mice were randomly divided into three groups (*N* = 10). All animals were subjected to bilateral ovariectomy (OVX) and then treated with saline (Veh), zoledronic acid (ZA), or concomitant zoledronic acid and dexamethasone (ZA/Dx) for 12 weeks. Eight weeks after starting drug administration, pulpal exposure was conducted on the lower left first molar. Four weeks after pulpal exposure, all mice were sacrificed and the mandibles were collected for radiological and histological examinations.

**Results:**

Microcomputed tomography (*μ*-CT) examination showed significantly reduced periapical bone resorption in the ZA/Dx group and decreased periodontal bone resorption in both ZA and ZA/Dx groups. Higher bone mineral density (BMD) and strengthened microstructure were found in ZA and ZA/Dx groups. More empty lacunae were found in ZA and ZA/Dx groups.

**Conclusions:**

Apical periodontitis aggravates MRONJ under immunocompromised circumstances. Concurrent use of ZA and steroids inhibits alveolar bone resorption but increases the risk of developing MRONJ.

## 1. Introduction

Medication-related osteonecrosis of the jaw (MRONJ) is an intractable clinical situation characterized by necrotic bone exposure in the oral cavity that does not heal for more than 8 weeks. Around 60–70% cases of MRONJ are associated with tooth extraction, where periapical diseases play a major role, resulting in the extraction. Recent clinical data show a potential link between periodontal/periapical diseases and MRONJ, which may explain the remaining 30–40% spontaneous MRONJ. The majority of MRONJ cases occur in cancer patients, who may have compromised immune status. Some cancers such as multiple myeloma require a concomitant course of dexamethasone and zoledronic acid (ZA). Considering the vast number of patients under bisphosphonates treatment and the high incidence of periapical diseases, any further understanding of the role of periapical infections in the development of MRONJ is of crucial clinical significance to prevent and treat this disease.

Higher incidence of MRONJ in cancer patients [1–3], who are frequently immunocompromised, has led to the hypothesis that dental infectious diseases under an immunocompromised health condition may trigger or exacerbate MRONJ.

We have recently developed a modified rodent model demonstrating intracortical and trabecular remodeling of the jawbones following a bilateral ovariectomy, mimicking the intracortical remodeling process of humans. The present study aimed to investigate the relevance of periapical diseases in MRONJ using an immunocompromised mouse model with ovariectomy.

## 2. Materials and Methods

### 2.1. Animal Care and Surgery

This study was approved by the Committee on the Use of Live Animals in Teaching and Research of the University of Hong Kong (CULATR 3084-13) and was performed in agreement with its guidelines. Thirty C57BL/6N female mice (20–25 g of weight, 11-12 weeks of age) used in this study were held and supervised by the LAU (laboratory animal unit) of Li Ka Shing Faculty of Medicine, the University of Hong Kong.

The animals were numbered and housed in 1144B cages (332.0 × 150.0 × 130.0 mm^3^) with filtered air and suitable temperature and humidity conditions in a pathogen-free facility with 12 h light/dark cycle. A standard diet of rodent chow and watered ad libitum was used to feed the animals. Acclimatization during a week prior to the beginning of the experiment was conducted, and animals which were considered to be in poor condition were excluded from the study. All animals were then subjected to bilateral ovariectomy (OVX) according to our previous study [4].

### 2.2. Study Design and Medication Treatment

Thirty C57BL/6N mice were randomly divided into three groups (*n* = 10). Six weeks after OVX surgery, saline solution (Veh), zoledronic acid (ZA), or concomitant ZA and dexamethasone (Dx) were administered to each group as depicted in Table 1. ZA was injected three times per week intraperitoneally (66 *μ*g/kg), and Dx was injected weekly subcutaneously (5 mg/kg). Saline solution was injected at the same intervals as experimental groups, both intraperitoneally and subcutaneously. The animals received consecutive injections for 8 weeks until pulpal exposure surgery and continued to receive injections for 4 weeks after this surgery.

### 2.3. Pulpal Exposure Surgery

After 8 weeks of injections, pulpal exposure procedures were performed on the lower left first molar of all animals under general anesthesia (ketamine HCl (100 mg/kg) and xylazine (5 mg/kg), i.p.). A ¼ round bur on a high-speed handpiece with saline irrigation for cooling was used to drill the tooth open and expose the pulp. The depth of the drilling was about the same as the diameter of the bur to avoid perforation of the furcal floor. An endodontic file (size 8) was used to remove the pulp tissue through the mesial and distal orifices. After the procedure, the tooth was left unrestored for the following 28 days. Postoperative analgesics were administered (Metacam® Boehringer-Ingelheim, Germany, 10 mg/kg, i.p.), and food/water intake and weight were monitored.

### 2.4. Sacrifice

After another 4 weeks of Veh, ZA, or ZA/Dx administration after operation, all animals were euthanized by intraperitoneal ketamine injections. The mandibles were then dissected and stored in 10% neutral buffered formalin solution for future examinations.

### 2.5. Clinical Examination

Signs of inflammation, infection, defective oral mucosa on alveolar bone, or sequestra were carefully examined and recorded.

### 2.6. Micro-CT Examination

**Figure 1:**
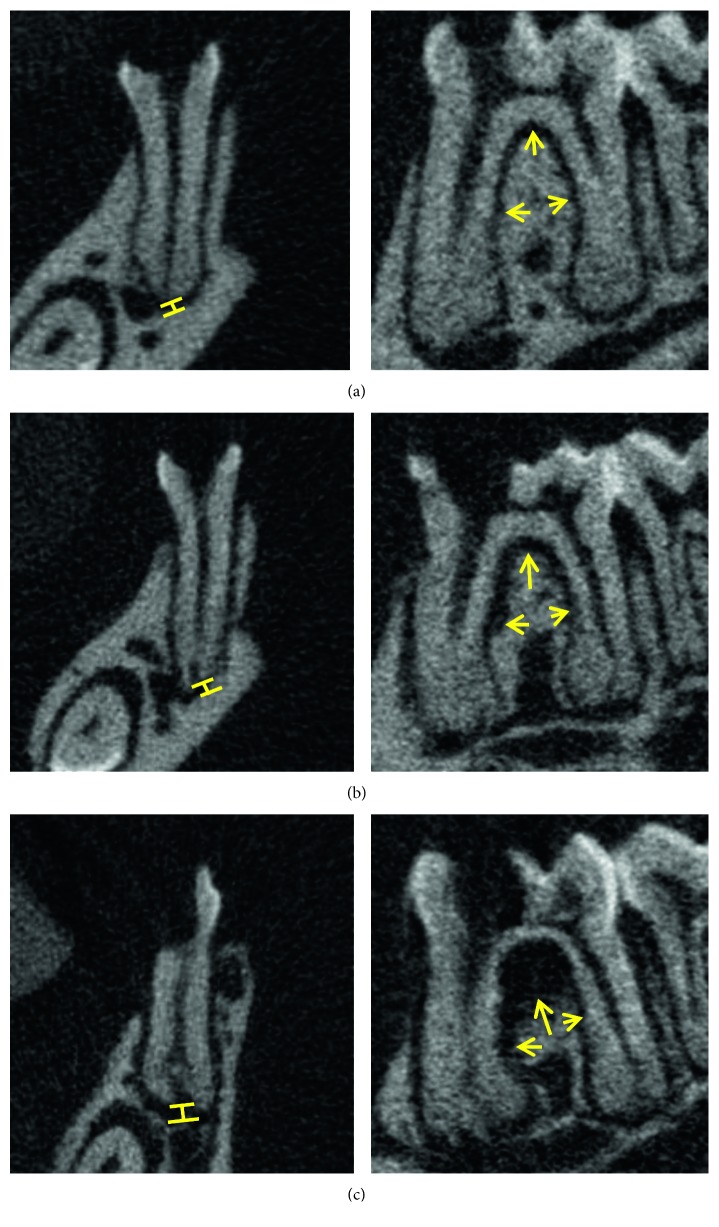
Periapical bone resorption (bars) and periodontal bone resorption (arrows) are observed among all three groups. Images on the left show periapical bone loss measurement. The images on the right show periodontal bone loss measurement. (a) ZA/Dx group. (b) ZA group. (c) Veh group.

Micro-CT scanning was performed using the Skyscan system according to the manufacturer's guidelines (voltage = 80 kV, current = 100 *μ*A, and exposure = 3993 ms). Images were scanned at a resolution of 8 *μ*m/pixel. Alveolar bone loss was assessed by measuring both periapical and periodontal bone loss of the operated tooth. Periapical bone loss was assessed by measuring the distance between the root apex and the periapical alveolar bone at the mesial root while periodontal bone loss was recorded by measuring the periodontal ligament width at 3 sites: the middle of the distal surface of the mesial root, the tip of the furcation, and the middle of the mesial surface of the distal root (Figure 1). Each measurement was conducted three times, and the average was recorded for each tooth. Other parameters such as bone mineral density (BMD) [5], bone volume/tissue volume (BV/TV), trabecular thickness (Tb.Th), trabecular number (Tb.N), and trabecular separation (Tb.Sp) were measured to assess bone quality and microstructure of the trabecular bone using CTAn software (CTAn 1.12.0, SkyScan) [6–8]. The region of interest (ROI) was selected at the interradicular septum of the first molar.

### 2.7. Histological Examination

Following micro-CT scanning, the specimens were decalcified in 14.5% EDTA solution (pH 7.2) at 25°C for 2 months and then dehydrated with graded ethanol (70%, 95%, 100%). After dehydration, specimens were embedded in paraffin and sectioned (5 *μ*m thickness) for histochemical examination [9], using hematoxylin and eosin (H&E) staining. Osteonecrosis was defined histologically as five contiguous empty osteocytic lacunae in trabecular bone in combination with osteocyte loss [10, 11]; 0 = no necrosis; 1 = necrosis. Quantitative analysis was done by randomly selecting four high power fields and calculating the incidence of osteonecrosis as in our previous study [10].

### 2.8. Statistical Analysis

Statistical analysis was performed with IBM SPSS statistics software (version 23.0, IBM Crop, Armonk:NY, USA). Independent *t*-test was performed to compare alveolar bone loss, bone density, and microstructure among each group, and the data were presented as mean ± SD. Fisher's exact test was used to compare the incidence of osteonecrosis. All tests were two sided, and a significance level of 0.05 was set.

## 3. Results

### 3.1. Clinical Examination

All animals completed the course uneventfully. No signs of inflammation, infection, or bone exposure were detected.

### 3.2. Micro-CT Analysis

**Figure 2:**
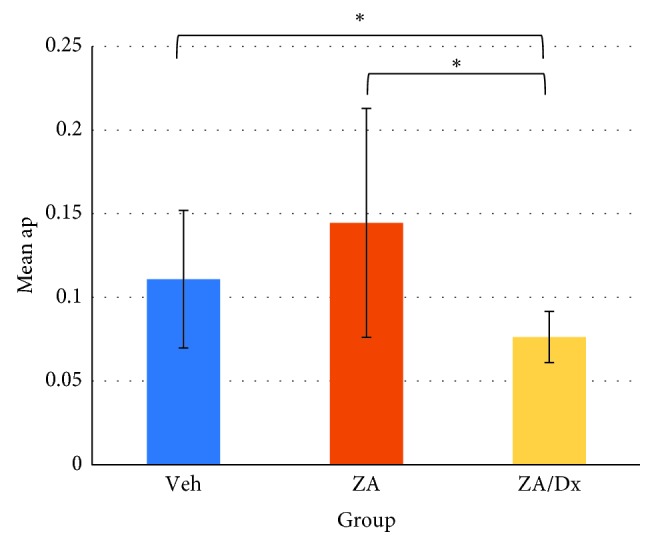
Effects of Veh, ZA, and ZA/Dx on periapical bone resorption. ap: root apex to periapical alveolar bone distance. ^*∗*^The difference is significant at level of *p* < 0.05.

**Figure 3:**
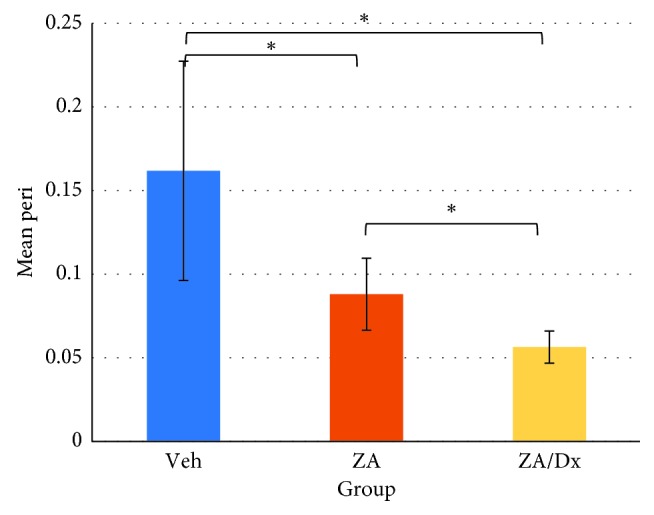
Effects of Veh, ZA, and ZA/Dx on periodontal bone resorption. Peri: periodontal ligament space width. ^*∗*^The difference is significant at a level of *p* < 0.05.

Alveolar bone loss in each group was compared using independent *t* test (Table 2). The ZA/Dx group showed significantly reduced periapical bone loss compared to the ZA group and Veh group (Figure 2). However, no significant difference was found between ZA and Veh groups (*p*=0.2). When comparing periodontal bone resorption, the ZA/Dx and ZA group exhibited remarkably less resorption than the Veh group, with the ZA/Dx group revealing significantly less resorption than the ZA group (Figure 3).

Drilled teeth were compared with their contralateral intact teeth to assess the effect of periapical disease on alveolar bone resorption. Significant periapical resorption was observed in both Veh and ZA groups (Table 3) and significant periodontal loss in all three groups (Table 4).

**Figure 4:**
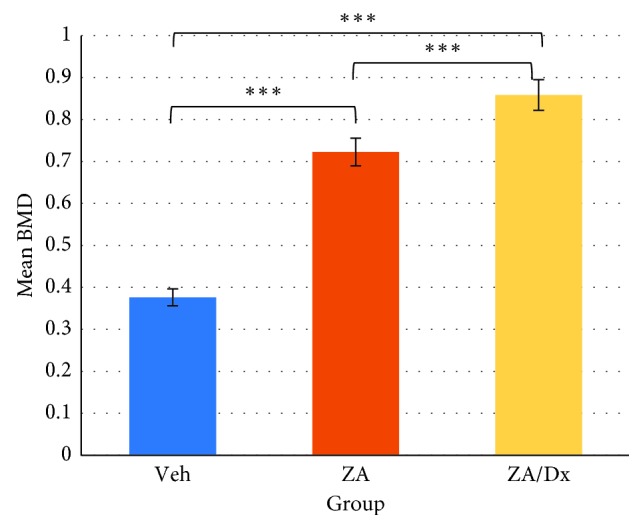
Effects of Veh, ZA, and Dx administration on BMD. ^*∗∗∗*^The difference is significant at level of *p* < 0.005.

**Figure 5:**
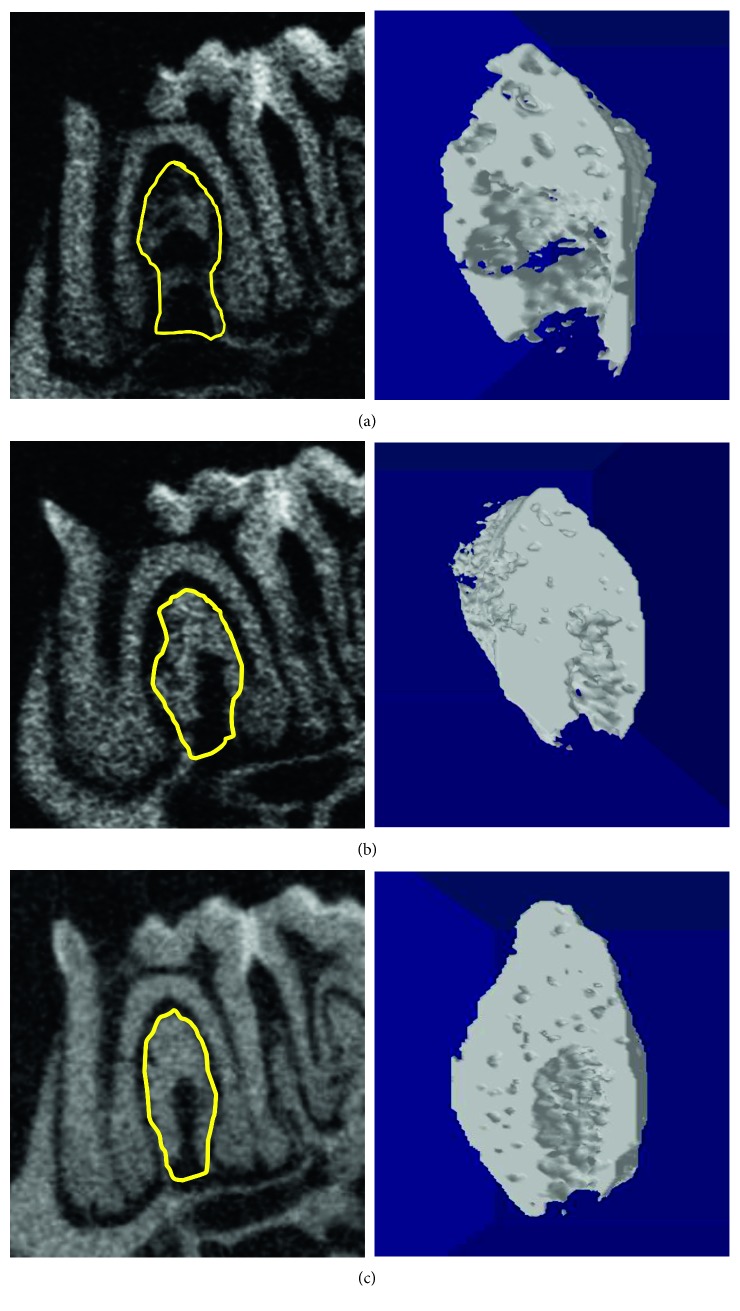
Micro-CT images for BMD analysis. The ROI in the trabecular bone for analysis is indicated by the yellow circle. (a) Vehicle group. (b) ZA group. (c) ZA/Dx group. Images on the right are the 3D images of the selected area.

BMD, BV/TV, Tb.Th, Tb.Sp, and Tb.N were compared between any two groups using independent *t* test (Table 5), before which the Shapiro–Wilks test and Levene's test were performed to assess normality and homogeneity, respectively. The ZA and ZA/Dx groups demonstrated significantly higher BMD compared to the Veh group (*p* < 0.0005, *p*=0.0111), with the ZA/Dx group showing markedly higher BMD than the ZA group (*p* < 0.0005) (Figures 4 and 5).

**Figure 6:**
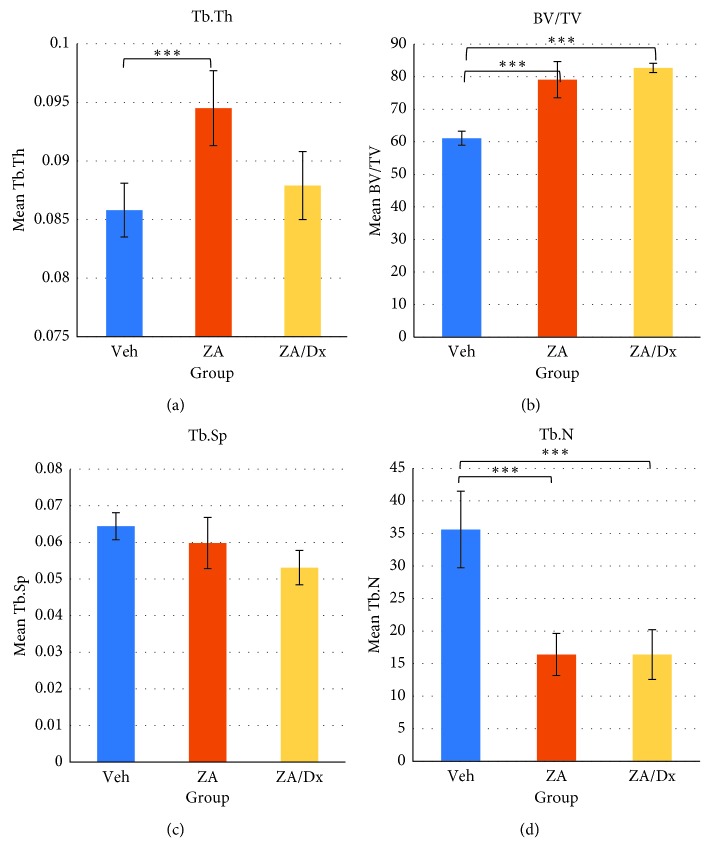
Effects of Veh, ZA, and Dx administration on alveolar bone microstructure. ^*∗∗∗*^The difference is significant at a level of *p* < 0.005.

Considerably increased BV/TV and Tb.Th and decreased Tb.N were observed in the ZA group compared to the Veh group (Figure 6). When comparing the ZA/Dx group with the Veh group, increased BV/TV and reduced Tb.N were detected (Figure 6).

### 3.3. Histological Examination

**Figure 7:**
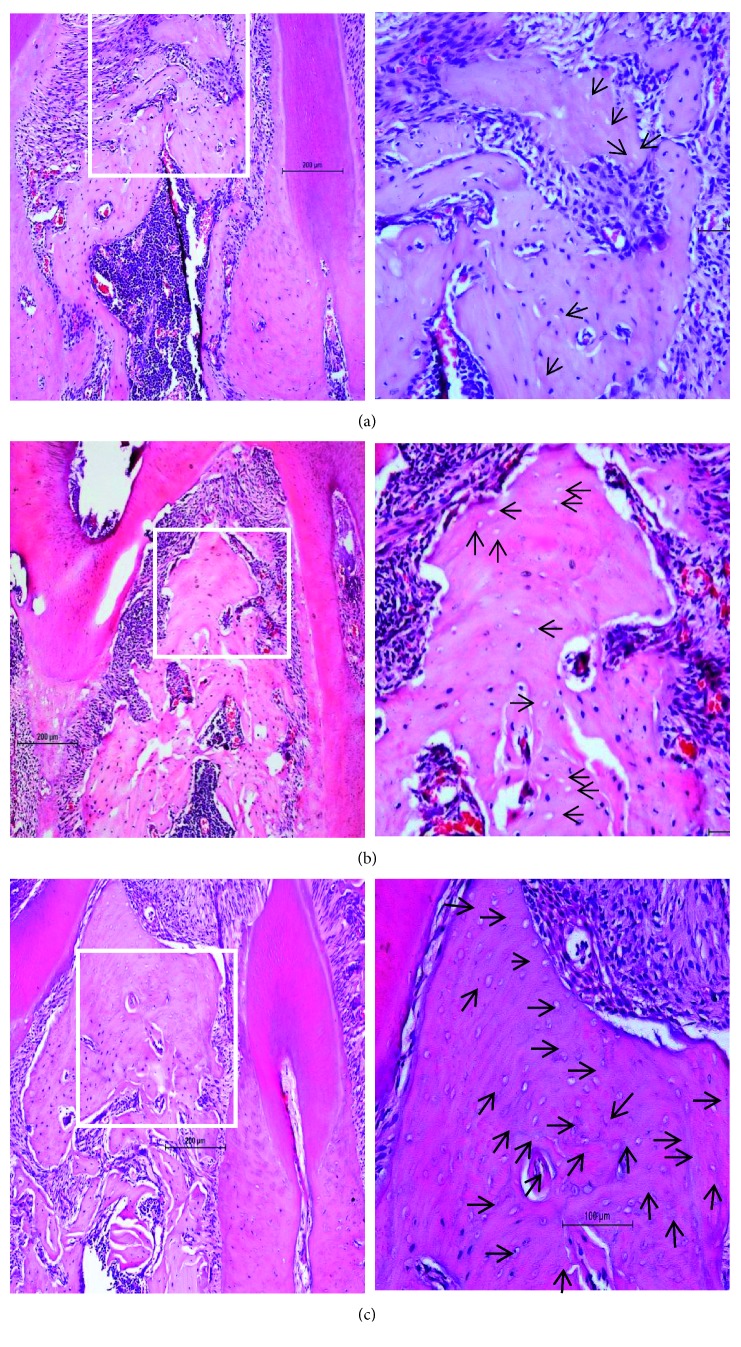
Histological images of the interradicular alveolar bone (H&E staining). (a) Vehicle group. (b) ZA group. (c) ZA/Dx group. More noticeable and extensive areas of lacunae and osteocyte loss were seen in the ZA and ZA/Dx groups, and the most noticeable accumulation of empty lacunae was found in the ZA/Dx group.

More noticeable and extensive areas of lacunae and osteocyte loss were seen in the ZA and ZA/Dx groups (Figure 7). Two cases of osteonecrosis were found in the ZA and ZA/Dx groups according to the histological necrosis criteria mentioned earlier. On the contrary, no necrosis was detected in the Veh group. Although no statistically significant difference in osteonecrosis incidence was found among groups (*p*=0.507), remarkably more empty lacunae were observed in mice administered ZA or ZA/Dx. Additionally, the most noticeable accumulation of empty lacunae was found in the ZA/Dx group (Figure 7).

Inflammatory response was also observed in areas adjacent to the interradicular bone where bone resorption occurred and in regions near osteonecrosis which is characterized by substantial amounts of empty lacunae (Figure 7). The inflammatory response can be categorized into acute inflammation and chronic inflammation. The acute inflammation was graded as mild (a few PMNs), moderate (small groups of PMNs), and intense (present in every microscopic field) according to the extent of PMN (polymorphonuclear neutrophils). The chronic inflammation shown as plasma cells and/or lymphocytes presenting in the specimen was also observed.

## 4. Discussion

For the investigation of MRONJ, the rodent model treated with bisphosphonates and steroids was considered reliable and reproducible [12, 13]. Dexamethasone, used in this study, is a synthetic member of the glucocorticoids group, which are highly potent anti-inflammatory agents. It exhibits an immunosuppressive potency of about 4-5 times of prednisone and 20–30 times of hydrocortisone [14]. 5 mg/kg body weight of dexamethasone was administered on a weekly basis to stimulate the immunocompromised condition. The dose of zoledronic acid was 66 *μ*g/kg body weight (three times per week), which is equivalent to the dosage for cancer patients treatment, based on the evidence that a cumulative higher dose implies a higher risk of developing MRONJ.

Periapical inflammation usually develops as the result of bacterial invasion in the root canal system [15]. Alveolar bone resorption was detected around the periodontal and periapical regions of the pulp-exposed teeth in all three groups, after sufficient time for periapical diseases is established. It is well acknowledged that infection can exacerbate bone resorption without the presence of osteoclasts, owing to the fact that direct bone resorption can be caused by bacteria and related fibroblast-like cells, releasing various acids and proteases [15]. This may well explain the resorption we observed in spite of the potent osteoclast-inhibitory effect of zoledronic acid. Such resorption, which is independent of osteoclasts, is likely to result in a lack of osteoblast-mediated bone formation which would take place in normal bone remodeling [16]. However, whether or not such osteoclast-independent bone resorption plays a role in the development of MRONJ still needs further investigation.

In this study, alveolar bone in the interradicular region exhibited more sensitivity in response to the inflammation than the periapical region. It is shown in Table 4 that an increase of approximately 3.6 times of the periodontal ligament width was detected in the pulp-exposed teeth compared to the contralateral intact teeth in the Veh group, whereas in the periapical area, the bone resorption of pulp-exposed teeth was only around 1.6 times of those nonexposed teeth (Table 3). In addition, systemic zoledronic acid either used alone or concomitant with dexamethasone demonstrated a pronounced effect on periapical bone protection. According to Metzger and et al. [15], dexamethasone exhibits the ability to significantly reduce the size of periapical lesions in rat models. These results indicate that steroids may affect bone resorption by downregulating the cytokine activity. However, concerns do exist upon the effect of steroids on inflammatory response such as apical periodontitis, since the downregulation of the host immune response may result in spreading of the root canal infection. Nevertheless, none of the animals treated with ZA/Dx exhibited any signs of infection spreading or localized periapical abscesses in this study.

Moreover, glucocorticoids exert an influence on bone metabolism by directly impeding osteoblast activity and indirectly stimulating osteoclasts through hormonal pathways, which is why glucocorticoids are used for rheumatoid arthritis (RA) patients, leading to an increased risk of osteoporosis. Corticosteroid-induced osteoporosis was reported to be the most common iatrogenic cause of secondary osteoporosis [17]. What makes our animal models more clinically pertinent is that RA patients are usually prescribed bisphosphonates to intercept bone loss. As our results showed, animals treated with concurrent zoledronic acid and dexamethasone exhibited a significantly higher BMD compared to those with zoledronic acid alone (*p*=0.011) and to those with vehicle saline (*p* < 0.0001). This indicates that there may be an intensified repressive effect of bisphosphonates on bone resorption, thus leading to an increased BMD when in combination with steroids. Concomitant administration of zoledronic acid and dexamethasone also resulted in strengthened bone microstructure in our study. Additionally, both ZA- and ZA/Dx-treated animals revealed significantly higher bone volume fraction (BV/TV) and lower trabecular number (Tb.N) than the control animals.

Aside from clinical and radiological examinations, the most reliable and powerful diagnostic method to confirm the existence of MRONJ is histological examination [18]. An analysis of the incidence of osteonecrosis was performed around pulp-exposed teeth. In one of our previous studies [19], we found a markedly elevated number of apoptotic osteocytes in periapical diseases, with or without zoledronic acid administration. In this study, continual empty lacunae were detected in both ZA-treated and ZA/Dx-treated mice, which indicates active subclinical micronecrosis has taken place. These histological osteonecroses were mainly detected adjacent to the interradicular region where evident bone resorption and inflammation were observed. As shown in the results, 20% of the ZA-treated and ZA/Dx-treated animals demonstrated osteonecrosis, whereas none of the control animals revealed that. Though the difference is not statistically significant, ZA/Dx administration did exert a magnified effect on inducing osteonecrosis compared to the control group, which is demonstrated by the remarkable aggravated condition of osteonecrosis. The situation is similar observing the ZA-treated group in comparison with the control group, and only the difference is smaller.

The use of immunosuppressive medication such as dexamethasone and other chemotherapeutic agents may lead to MRONJ because of their anti-inflammatory effects, which might cause an immunocompromised condition in some patients [9, 12]. Numerous researches have been done to study the relationship between immunosuppressive drugs and MRONJ [20, 21]. Bi et al. [9] published a 100% incidence of MRONJ in both ZA-treated and ZA/Dx-treated groups induced by tooth extractions and various medication treatments. Nevertheless, greater severity of the lesions and larger necrosis regions were detected in the ZA/Dx-treated animals. The findings in this study were also in accordance with recent research that states that zoledronic acid used alone does not differ from concurrent use of steroids and zoledronic acid on the occurrence of MRONJ [12]. In contrast, Sonis et al. [20] reported that MRONJ lesions were found following tooth extractions in the ZA/Dx group only; neither ZA alone or Dx alone animals found such lesions.

Normally, physiologic osteocyte apoptosis processes also exist, yet they can be well modulated and balanced through the bone remodeling process. The omnipresent accumulation of dead osteocytes in MRONJ was considered the result of medication, bisphosphonates, for instance, suppressing bone remodeling, in which case nonviable osteocytes could not be eliminated by impaired remodeling processes; therefore, accumulation can be expected. However, this speculation cannot answer all questions regarding the pathogenesis of MRONJ, for example, why ONJ only affects the jawbones.

Considering their close relation with the jawbones, microorganisms and odontogenic infectious diseases may play a role in the development of MRONJ. As we had discussed above, microbial colonies were frequently identified in samples of MRONJ patients [22]. In addition, apical periodontitis is essentially an immune-mediated host response, precipitated by microbial colonization, easily spreading into the surrounding jawbones. Therefore, the conjecture that apical periodontitis contributes to the development of ONJ exists; thus, this disease was implanted in our study. On the other hand, high dosage of bisphosphonates equivalent to the dose used for cancer patients was implemented, along with a potent immunosuppressive agent, dexamethasone. Consequently, an incidence as high as 20% of subclinical MRONJ in ZA and ZA/Dx groups was demonstrated in this study, which is much higher than that seen in human beings. The character of periapical infection in the development of MRONJ may be elucidated as follows. First of all, after a sufficient period of time as well as a stable concentration of bisphosphonates has been achieved, the jaw bone remodeling process slowed down, resulting from the antiremodeling therapeutic effect of these drugs. It was reflected in the results of this study showing that bisphosphonates with or without the concurrent use of steroids could result in a significantly higher BMD and a strengthened microstructure. Secondly, bacterial infection triggered responses in the immune system, elevating the extent of inflammation. As a consequence, an escalated bone turnover rate would be required for healing to take place. Moreover, glucocorticoids are thought to enhance microbial infection due to their immunosuppressive effect. The disharmony between the demand to heal and the impaired bone remodeling process may therefore lead to the cumulation of osteonecrosis and eventual bone exposure [23].

## 5. Conclusion

This study provides solid evidence that MRONJ is exacerbated by apical periodontitis in an immunocompromised condition. Concomitant zoledronic acid and steroids inhibit alveolar bone resorption but increase the risk of developing MRONJ.

## Figures and Tables

**Table 1:** Animal groups and medication administration.

Group	Number of animals	Medication	
		ZA	Dx
Veh	10	−	−
ZA	10	+	+
ZA/Dx	10	+	+

Veh: vehicle saline. ZA: zoledronate acid. Dx: dexamethasone.

**Table 2:** Alveolar bone resorption of the lower PE molar.

Group	Medication	Number	AP ± SD (mm)	PDL ± SD (mm)
Veh	ZA(−)/Dx(−)	10	0.1109 ± 0.0411	0.1618 ± 0.0655
ZA	ZA(+)/Dx(−)	10	0.1445 ± 0.0684	0.0880 ± 0.0215
ZA/Dx	ZA(+)/Dx(+)	10	0.0763 ± 0.0153	0.0564 ± 0.0096

AP: root apex to periapical alveolar bone distance. PDL: periodontal ligament space width.

**Table 3:** Periapical bone resorption between PE and non-PE teeth.

Groups	Medications	Non-PE tooth ± SD	PE tooth ± SD	*p* value
Veh	ZA(−)/Dx(−)	0.0705 ± 0.0208	0.1109 ± 0.0411	0.013
ZA	ZA(+)/Dx(−)	0.0787 ± 0.0309	0.1445 ± 0.0684	0.012
ZA/Dx	ZA(+)/Dx(+)	0.0695 ± 0.0259	0.0763 ± 0.0153	0.481

**Table 4:** Periodontal bone resorption between PE and non-PE teeth.

Groups	Medications	Non-PE tooth ± SD	PE tooth ± SD	*p* value
Veh	ZA(−)/Dx(−)	0.0445 ± 0.0064	0.1618 ± 0.0655	<0.0001
ZA	ZA(+)/Dx(−)	0.0418 ± 0.0114	0.0880 ± 0.0215	<0.0001
ZA/Dx	ZA(+)/Dx(+)	0.0420 ± 0.0103	0.0564 ± 0.0096	0.005

**Table 5:** Effect of Veh, ZA and ZA/Dx on BMD and bone microstructure.

Group	Medication	Number	BMD	BV/TV	Tb.Th	Tb.Sp	Tb.N
Veh	ZA(−)/Dx(−)	10	0.3761	61.1034	0.0858	0.0644	35.6
ZA	ZA(+)/Dx(−)	10	0.7228	79.0763	0.0945	0.0598	16.4
ZA/Dx	ZA(+)/Dx(+)	10	0.8585	82.6860	0.0879	0.0531	16.4

## Data Availability

The data supporting the findings of this study are included within the article.
